# Cost analysis of two community-based HIV testing service modalities led by a Non-Governmental Organization in Cape Town, South Africa

**DOI:** 10.1186/s12913-017-2760-8

**Published:** 2017-12-02

**Authors:** Sue-Ann Meehan, Nulda Beyers, Ronelle Burger

**Affiliations:** 1 Desmond Tutu TB Centre, Department of Paediatrics and Child Health, Faculty of Medicine and Health Sciences, Stellenbosch University, Francie van Zijl Ave, Parow, Cape Town, South Africa; 2 Department Economics, Stellenbosch University, Cape Town, South Africa

**Keywords:** Cost analysis, Community-based, HIV testing services, Mobile, Stand-alone, NGO

## Abstract

**Background:**

In South Africa, the financing and sustainability of HIV services is a priority. Community-based HIV testing services (CB-HTS) play a vital role in diagnosis and linkage to HIV care for those least likely to utilise government health services. With insufficient estimates of the costs associated with CB-HTS provided by NGOs in South Africa, this cost analysis explored the cost to implement and provide services at two NGO-led CB-HTS modalities and calculated the costs associated with realizing key HIV outputs for each CB-HTS modality.

**Methods:**

The study took place in a peri-urban area where CB-HTS were provided from a stand-alone centre and mobile service. Using a service provider (NGO) perspective, all inputs were allocated by HTS modality with shared costs apportioned according to client volume or personnel time. We calculated the total cost of each HTS modality and the cost categories (personnel, capital and recurring goods/services) across each HTS modality. Costs were divided into seven pre-determined project components, used to examine cost drivers. HIV outputs were analysed for each HTS modality and the mean cost for each HIV output was calculated per HTS modality.

**Results:**

The annual cost of the stand-alone and mobile modalities was $96,616 and $77,764 respectively, with personnel costs accounting for 54% of the total costs at the stand-alone. For project components, overheads and service provision made up the majority of the costs. The mean cost per person tested at stand-alone ($51) was higher than at the mobile ($25). Linkage to care cost at the stand-alone ($1039) was lower than the mobile ($2102).

**Conclusions:**

This study provides insight into the cost of an NGO led CB-HTS project providing HIV testing and linkage to care through two CB-HIV testing modalities. The study highlights; (1) the importance of including all applicable costs (including overheads) to ensure an accurate cost estimate that is representative of the full service implementation cost, (2) the direct link between test uptake and mean cost per person tested, and (3) the need for effective linkage to care strategies to increase linkage and thereby reduce the mean cost per person linked to HIV care.

**Electronic supplementary material:**

The online version of this article (10.1186/s12913-017-2760-8) contains supplementary material, which is available to authorized users.

## Background

South Africa has a large burden of human immunodeficiency virus (HIV); approximately 6.4 million people are infected with HIV [1] and 3.1 million are on treatment (2014/15) [2]. The financing and sustainability of HIV services have become a priority [3]. In response to the HIV epidemic, South Africa has adopted the UNAIDS “90–90-90” target; by 2020, 90% of all people living with HIV should know their status, 90% of all eligible people with diagnosed HIV infection should receive sustained antiretroviral therapy (ART), and 90% of all people receiving ART should have viral load suppression [4]. There are limited data on population level of people aware of their HIV status – in one district in South Africa, it has been estimated that 48% of men and 35%of women are unaware that they are living with HIV [5]. HIV testing services (HTS) have a pivotal role to play in the pursuit of the first “90” by expanding the proportion of people living with HIV who know their status.

Taking HIV services closer to beneficiaries and communities to improve test uptake [6] is important. Community-based HIV testing services (CB-HTS) can reach populations who do not typically access health facilities, for example, males [7–9], making them a viable alternative to government-led facility-based services. Community-based services can provide HIV testing: (1) on a mobile basis (using mobile vans and tents) which can reach more men compared to facility-based services [10, 11]; (2) at stand-alone facilities (fixed sites), which have proportionately more clients who test HIV-positive compared to mobile services [12]; and (3) in the home, which reaches more first-time testers and males compared to mobile services [13].

The current trend in sub-Saharan Africa is to outsource community-based services to non-governmental organizations (NGOs) [14], making it vital to understand the impact that NGO-led HIV testing services can have in reaching those unaware of their HIV status. While CB-HTS have been shown to be feasible [15] and acceptable [16], there is limited literature on the cost of implementing and maintaining NGO-led CB-HTS, or on what drives the cost of NGO-led services. This is important for future planning to ensure the sustainability of these services, especially within the context of declining donor funding [3] and the continuing need to scale up CB-HIV testing services.

As CB-HIV testing modalities may differ in their infrastructure (e.g. fixed site or mobile) and client characteristics (proportion of males, first-time testers, HIV-infected), it is important to estimate the cost of each modality. This study therefore explored the costs of implementing and providing services at two community-based NGO-led HIV testing modalities, stand-alone and mobile, in Cape Town, South Africa. It also calculated the mean cost associated with key HIV outputs (HIV testing, diagnosis and linkage to HIV care) for each modality.

## Methods

### Setting

This study took place in the Cape Metro district, Western Cape Province, South Africa. This district, home to 66% of the Province’s population [17], has an antenatal HIV prevalence of 20.4% [18].

This study was embedded within a large community-based HIV testing services (HTS) project. The Desmond Tutu TB Centre (DTTC) at Stellenbosch University worked in partnership with five non-governmental organizations (NGOs) in five peri-urban communities within the Cape Metro district. These communities were all characterized by poverty, overcrowding, high unemployment rates, and high HIV prevalence [19]. Each NGO worked in a separate community, where they each implemented two community-based HIV testing modalities: a stand-alone center and a mobile service. The analysis was conducted with data from one NGO.

### Description of the CB-HTS project

Stellenbosch University awarded each NGO a contract to provide CB-HTS via a tender process, with successful NGOs demonstrating good financial and management capacity, as well as relevant experience working in high-HIV-burden communities around Cape Town. Stand-alone HTS centers were fixed sites in accessible locations that allowed clients to walk in without an appointment and request any of the services provided as part of a comprehensive “HTS service package”. Mobile services consisting of “pop-up” tents and a caravan (mobile van) were set up in the community near transport hubs, on open fields, or next to main thoroughfares. These sites were selected on an ad hoc basis by the HTS team and changed regularly. Some sites were visited more than once if there was sufficient demand for services at a particular site. Services at the stand-alone and the mobile sites were the same and were delivered using standard operating procedures. Services included HIV testing, TB and STI symptomatic screening, TB testing, screening for non-communicable diseases, pregnancy testing, assessment of family-planning needs, general health education, and linkage to relevant care and treatment services. Clients who did not want an HIV test could still access any of the other services provided.

While NGOs were responsible for service implementation, DTTC was responsible for the overall management of the CB-HTS project, as well as ensuring contractual obligations to the funder, providing technical assistance to the NGOs (training, mentoring and funding), data management, and overall financial management. DTTC monitored NGO expenditure and progress toward targets on a quarterly basis through the timely submission of financial and narrative reports by each NGO.

For each NGO, the core staffing complement was identical and consisted of a coordinator, a professional nurse, an enrolled nurse, and three trained HIV lay counsellors. The day-to-day logistics, management, and monitoring and evaluation of services were the responsibility of the coordinator and the professional nurse. The enrolled nurse provided the majority of the clinical services under the supervision of the professional nurse [20], who provided clinical services when required. The HIV lay counsellors provided pre- and post-test counselling and HIV rapid testing. The nurses were employed by Stellenbosch University, but seconded to the NGO. The coordinator and HIV lay counsellors were employed directly by the NGO. Support personnel, based at DTTC and the NGO offices, were tasked with overseeing the management, operations, human resources and data aspects of the CB-HTS project. Support personnel were employed to perform a number of other duties at DTTC and the NGO unrelated to this project and hence gave a proportion of their time to this project. (See Additional file 1 for categories of core and support personnel.)

A bi-annual audit^1^ monitored and evaluated a standardized quality service across all five stand-alone centers and mobile services, and also between services provided at stand-alone centers and on a mobile basis. This was to ensure that clients received an identical service irrespective of which HTS modality they accessed.

### HIV testing services

After pre-test counselling, clients voluntarily consented to an HIV test. HIV testing was done in accordance with provincial algorithms and guidelines. Using a serial testing algorithm, an HIV diagnosis was made when both the screening (Advanced Quality HIV-1/2) and confirmatory (Abon HIV-1/2/0 Tri-line) rapid tests were positive. Discordant results were confirmed with a laboratory ELISA (Enzyme-Linked Immunosorbent Assay) test performed by the National Health Laboratory Service (NHLS). Clients received their HIV test result during post-test counselling. HIV-positive clients were given a referral letter to a public health facility for HIV care and treatment. In addition, they were followed up telephonically to determine self-reported linkage to care. At least three attempts over a 3-month period were made to contact the client to determine linkage to care. During the telephone interviews clients were asked a number of relevant questions such as: Where did you access HIV care? Who did you see? What were you told? When is your next appointment? If the individual could answer each question, and provide adequate detail, they were considered to have linked to care and were documented as such. If the client could not be reached telephonically after a number of attempts at various times of the day, then a home visit was done.

### Selection of study site

We purposively selected one of the five communities in which the CB-HTS project was implemented. Across all five communities (1) socio-economic circumstances (peri-urban areas described above) were similar; (2) services were implemented with the same number of personnel and personnel categories; (3) remuneration was similar for same-category personnel; (4) identical services were provided within the same standard operating procedure framework; (5) personnel efficiency was similar, i.e., similar numbers of clients were provided with services and a similar number of quality assurance practices and monitoring and evaluation practices were completed; and (6) public primary health care facilities existed where HTS is offered, providing an alternative venue for accessing HTS for those living in these communities. The community selected for the study was chosen because of the long standing working relationship between DTTC and the NGO working in that community.

### Cost data collection

As the NGO provided services, this study used a service provider perspective, considering the resources utilized and the HIV outputs achieved in the implementation of two CB-HIV testing modalities. We obtained quarterly project costs for the period July to September 2014. Assuming similar costs per quarter, these costs were then annualized by multiplying each quarterly cost by four. In addition, the annual financial expenditure report for this CB-HTS project was used to identify any additional costs, which were also listed. These included, for example, annual costs (insurance and occupational health cover) and ad hoc costs such as the printing and purchasing of Information, Education and Communication (IEC) materials. The instrument for collecting data was adapted from a costing tool developed by the International Training and Education Centre for Health (I-TECH) [21, 22] and developed in Microsoft Excel 2011. All costs were calculated in South African rands (ZAR) and then expressed in 2014 US dollars (exchange rate R11/$1).

Costs were collected from three sources: (1) the NGO (costs incurred directly by the NGO with funds secured from DTTC including salaries, office furniture and medical consumables); (2) DTTC (costs incurred directly at DTTC such as support personnel salaries, computers used by support personnel, and printing); and (3) the Provincial Department of Health (HIV test kits and condoms were supplied free of charge to the project).

### Measurement of costs

The economic costs of implementing two CB-HTS modalities were calculated using an ingredients approach. All inputs were identified, measured, and allocated.

#### Overview of allocation of costs

Firstly, costs were divided into three broad categories: personnel (divided into core and support personnel), capital goods, and recurring goods and services. Secondly, we considered how each of these broad cost categories contributed to seven project components: (1) administration, (2) capacity-building (training/coaching/mentoring), (3) monitoring and evaluation, (4) data, (5) planning, (6) direct service provision, and (7) overheads.

To compare cost per modalities, we allocated shared costs across the mobile and stand-alone modalities. We used client volumes and personnel time as the allocation base. As some costs were driven by personnel share and other costs were driven by client volumes, we used both allocation bases. Costs such as salaries, security, and occupational health care are associated with personnel and were allocated based on the share of personnel time devoted to each modality. Costs such as HIV rapid test kits, IEC materials, and medical waste disposal are closely associated with client volumes and were allocated using the modalities’ client volume share. (See Additional file 2 for examples of costs included in each project component per cost category, per HTS modality.)

#### Cost categories

##### Core personnel costs

All core personnel (*n* = 6) in the study site consented to keep activity sheets for the period July to September 2014. These activity sheets recorded their time spent per project component at either the stand-alone center or the mobile service. They recorded their activities at 30-min intervals throughout the day, using pre-determined coding. A data clerk entered the data from the activity sheets into a Microsoft ACCESS 2013 database which was developed specifically for this study. Where time slots were blank, this was coded as such in the database. The database was used to determine the proportion of time spent per project component within each HTS modality for each core personnel individual. Personnel remuneration was received from the human resources department and from NGO salary advices. Summaries of personnel time, together with their salary costs, were imported into the Excel spreadsheet.

##### Support personnel costs

All identified support personnel (*n* = 10) provided written informed consent to be interviewed. The interviews were conducted between March and September 2015 and followed a pre-determined template. Support personnel identified each activity that they performed within the CB-HTS project. The costs for each support person were entered into the Excel spreadsheet as overheads, proportionately allocated across the two HTS modalities.

##### Capital goods

The equipment inventories at the NGO and DTTC were used to compile a list of capital goods. For each item the year of purchase, unit price, quantity, and the useful life years were captured. It was noted that six kinds of items made up 80% of the total cost of capital items (one mobile van, one laptop, three large tents, two small tents, two point-of-care CD4 analyzers, and a proportion of the database server). Assuming depreciation remained constant each year, and using an interest rate of 8% [23], the equivalent annual cost for each of these six items was calculated and converted to 2014 prices using the South African consumer price index (CPI) rate [24] for the year in which the item was purchased. The 20% remaining balance of smaller capital item costs was calculated based on the presumption that these items had the same structure. For these items we used an average CPI rate. The cost of the capital items was then apportioned across the HTS modalities and across the project components within each modality.

##### Recurring goods/services

The cost data for all recurring goods/services was gained from invoices paid, either by DTTC or by the NGO. We used the NGO financial quarterly report (July to September 2014) for cross-checking, to ensure all recurring goods/services were included as well as the overall CB-HTS project financial expenditure report to ensure that costs incurred annually, e.g., mobile van licensing and servicing, were included. The Provincial Department of Health provided costs for HIV test kits and condoms, which were included in the study although they were provided free of charge to the project. The costs were allocated appropriately between the seven project components and across the two HTS modalities.

### HIV outputs

The HIV outputs measured in this study included (1) the number of people who received pre-test counselling (which includes a package of related services described above), (2) the number of people who consented to and had a screening HIV rapid test, (3) the number of people who were diagnosed as HIV positive (according to the HIV testing algorithm), (4) the number of HIV-infected people referred for HIV care, and (5) the number of people who self-reported that they had linked to HIV care. These outputs were measured for those who attended HTS at the stand-alone center and the mobile services between January and December 2014. We assessed a longer time series of data to confirm that the period July to September was not an outlier, but provided a representative picture of HIV outputs for this NGO.

### Data analysis

We calculated the total cost of each HTS modality and the cost categories (personnel, capital, and recurring goods/services) across each HTS modality. Costs per project component were calculated to examine cost drivers overall and per HTS modality. HIV outputs were analyzed and then the mean cost for each HIV output was calculated per CB-HIV testing modality based on total cost of the modality divided by the specific HIV output. (For example, the mean cost per person tested at stand-alone = total cost of stand-alone service modality/total number of people tested at stand-alone.)

### Ethics approval

The study was approved by the Health Research Ethics Committee of Stellenbosch University (S12/02/059). A memorandum of understanding exists between Stellenbosch University, the Cape Town City Health Directorate and the Provincial Department of Health regarding the implementation of CB-HTS. All personnel (employed by Stellenbosch University and the NGO) who either completed a time allocation sheet or who were interviewed, provided written informed consent. The NGO also provided written informed consent to use its quarterly financial report. No incentives were given to any study participant or client who tested for HIV.

## Results

### Overall costs per cost category

The overall annual cost of the CB-HTS delivered by the NGO selected for this analysis was $174,380, with stand-alone costing $96,616 and mobile services $77,764. Of the three cost categories, personnel costs accounted for 50% of the total costs (Table 1). For the stand-alone modality, personnel costs accounted for the highest proportion of costs (54%). Within the mobile modality, the proportion of costs spent on personnel and recurring goods/services costs were both 46%. Capital costs were higher for the mobile modality than for the stand-alone.

**Table 1:** Overall costs per cost category per CB-HTS modality

	Stand-Alone	% of stand-alone cost	Mobile	% of mobile cost	Total	% of total cost
Personnel	$51,715	54%	$35,166	46%	$86,881	50%
Capital goods	$4358	5%	$6740	8%	$11,098	6%
Recurring goods/services	$40,543	41%	$35,858	46%	$76,401	44%
Total	$96,616		$77,764		$174,380	

Personnel included costs of all core and support personnel

Capital goods included costs of all equipment and assets

Recurring goods/services included costs of all utilities, consumables and services directly related to the HIV testing services

### Costs per project component

**Fig. 1:**
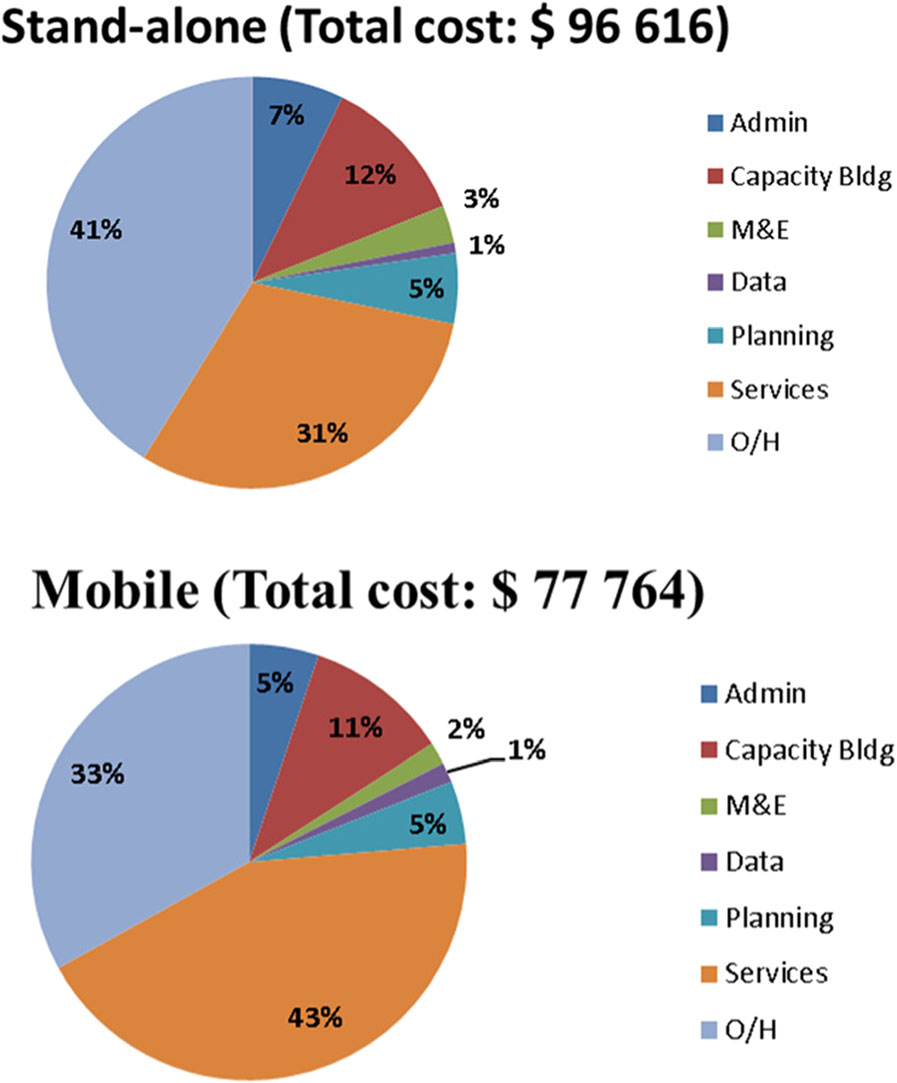
The proportion of costs per program component per CB-HIV testing modality

Overall, overheads and service provision accounted for the majority of the expenditure (37% and 36% respectively). Capacity-building made up 11% of the total costs, while administration, monitoring and evaluation, data, and planning each made up less than 7% of the total costs. As seen in Fig. 1, at the stand-alone modality, overheads were the main cost driver, accounting for 41% of the overall cost. Within the mobile modality, service provision accounted for a higher proportion of the expenditure (43%) than overheads (33%).

**Fig. 2:**
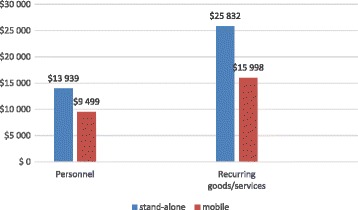
Overhead costs per cost category per modality

Overheads accounted for 27% of costs within the personnel category (which included salaries of support personnel) and more than half (55%) of costs within the recurring goods/services category (which included rental, utilities, telephone, cleaning, security, and NGO administrative costs (not shown). Figure 2 shows that overhead costs in both the personnel and recurring goods/services categories at the stand-alone center were higher than at the mobile.

### HIV outputs and costs

Overall, 5031 clients accessed the NGO-led CB-HTS in the study site between January and December 2014, of which 3104 (61%) accessed the mobile modality. The median age of individuals was 24 years (IQR: 17–33). A lower proportion of males (26%) utilized the stand-alone modality than the mobile modality (55%). As shown in Table 2, the majority of clients consented to an HIV test (98%) irrespective of which modality they accessed. Of those who tested for HIV, a higher proportion were diagnosed HIV-positive at the stand-alone centers (6%) than at the mobile services (2%). Self-reported linkage to care was higher for those who accessed stand-alone centers (74%) than at mobile services (50%).

**Table 2:** HIV outputs and cost per HIV output per CB-HIV testing modality

	TOTAL	Stand-alone $ 96,616		Mobile $ 77,764	
		Number	Cost per person	Number	Cost per person
Counselled	5031	1927	$50	3104	$25
Tested (%)	4966 (98)	1909 (99)	$51	3057 (98)	$25
HIV diagnosed (%)	202 (4)	128 (6)	$755	74 (2)	$1051
HIV referred (%)	198 (98)	125 (97)	$773	73 (98)	$1065
Linked to HIV care (%)	130 (65)	93 (74)	$1039	37 (50)	$2102

The overall mean cost per person counseled and tested at stand-alone centers ($51) was higher than at the mobile modality ($25). At the stand-alone, the mean cost of diagnosing a person with HIV ($755) and of successfully linking an HIV-infected person to HIV care ($1039) was lower than at the mobile. See Table 2.

## Discussion

This study provides insight into the cost for an NGO-led CB-HTS, providing HIV testing and linkage to care through two modalities. Overall, the stand-alone modality cost more than the mobile modality. Personnel costs accounted for half of the total cost overall and 54% of the cost of the stand-alone modality. These findings are consistent with other studies which found personnel costs to be a cost driver [15, 25, 26]. Labor costs are high in African countries (including South Africa) compared to similar economies in Asia [27]. This highlights the need for good management of personnel and their activities. Continually improving managerial skills is critical, as these skills may be lacking in the health sector, where clinically trained personnel typically become managers.

Costs for capital goods and recurring goods/services as a proportion of the total spent at each HIV testing modality were higher for the mobile modality than the stand-alone. This was predominantly due to the mobile van and tents that were used exclusively for mobile services and the higher number of medical goods/services required at mobile services due to the greater numbers of people accessing the mobile modality.

Of interest is that costs associated with service provision represented less than half the total costs at both modalities. This was predominantly due to the high overhead costs that can be associated with the manner in which this project was implemented (cost of managing the project at DTTC and the NGO). The overhead costs were comprehensive and directly associated with ensuring quality within the project. Reducing these overheads would have resulted in lower levels of management (general and data) together with fewer monitoring and evaluation systems and continual training and coaching. All of these aspects are important for quality service provision. Future work is required to understand the relationship between quality of services and overheads in more detail.

Fewer individuals accessing counseling and testing at the stand-alone modality resulted in a higher cost per person counseled and tested at the stand-alone modality ($51) compared to the mobile modality ($25). This cost comparison is similar to that reported in previous publications [6, 28]. Compared to the mean cost per person tested in other studies, our findings show a similar cost for the mobile modality [11, 15], but a higher cost at the stand-alone modality [28–30]. Future studies can look at ways of improving personnel efficiency and increasing test uptake. Based on the analysis here it is likely that higher client volumes and diverting more HIV testing paperwork to administrative personnel could reduce the mean cost per person counseled and tested.

Mobile services achieved lower costs per person tested, making mobile services an important strategy to consider when working toward achieving the first “90”. However, the benefits of increased testing uptake need to be balanced against the yield of HIV-positive cases. The mobile modality had a lower proportion of individuals who were diagnosed with HIV and who reported successful linkage to care than the stand-alone modality. These lower yields resulted in the cost per person diagnosed and successfully linked to HIV care being higher at the mobile modality than at the stand-alone modality. Future studies should look at evidence-based interventions to improve linkage to care, especially from mobile services. It is also important to highlight that HIV testing services can link HIV-negative individuals to HIV prevention interventions, for example voluntary male medical circumcision (VMMC) or pre-exposure prophylaxis (PreP). This may improve cost efficiency at HIV testing services and should be further investigated.

Our results showed that the mobile modality was utilized by a greater proportion of males, which is important given that this is a subpopulation that has been more difficult to reach. The cost of a CB-HTS modality should be considered together with the ability of that modality to reach certain ‘hard to reach’ populations e.g. males. This is specifically true for the mobile modality. Future studies can consider costing around reaching a range of subpopulations that have been shown to be difficult to reach through traditional HIV testing avenues.

The costs of diagnosing and linking individuals to care within this project were much higher than has previously been reported for South Africa [6] and in Swaziland [15]. The current HIV environment within South Africa (general decline in HIV incidence) [1] may have contributed to higher costs in finding HIV-infected individuals. Many published studies used data obtained prior to 2010 when fewer South Africans had tested for HIV. Since the launch of the National HTS campaign in 2010, 35 million South Africans have tested [2]. With more people aware of their HIV status, targeted approaches are now required to achieve the first “90” in the most cost-effective manner. Future costing studies will need to determine the costs of CB-HTS strategies that are implemented in different ways, while ensuring a comprehensive accounting of overhead costs, to accurately define the cost of finding and diagnosing individuals with unknown HIV.

Shared resources and economies of scale reduce individual project costs [31]. This should be considered when comparing the costs of this project with those of other HIV testing services in South Africa. In this NGO-led project, the specific clinical activities resulted in lower utilization of personnel compared to a government health facility scenario, where the range of services is broader and personnel work across services. This reflection provides some understanding of the kinds of costs that drive expenditure for an NGO-led CB-HTS project. The South African rand (ZAR) has weakened considerably against the US dollar since 2004. As costs are collected in ZAR and expressed in USD, exchange rate fluctuations, together with an undervalued South African rand [32], should be considered when comparing the expenditure of this project to other South African CB-HTS.

A major strength of this study is that it provides a thorough example of the costs of testing for HIV within an NGO-led project providing CB-HTS across two HTS modalities within a constrained health care setting. The first author had access to all costs, resulting in a realistic assessment of the costs involved in implementing CB-HTS for the stand-alone and mobile modalities. This is in contrast to other costing studies, where retrospective data collection may have been subject to recall bias [33] or poor record-keeping [25]. The data collection process was intensive and detailed, allowing for an in-depth and realistic understanding of the proportion of core personnel time across project components. A further strength is that this study includes more recent cost data than existing published studies.

As this study was conducted from a service provider (NGO) perspective, we acknowledge the exclusion of patient costs, (typically costs associated with travel and waiting time), as a limitation. However, we do not believe that patient costs would have been different for mobile and stand-alone modalities, based on prior (unpublished) work by the first author. A second limitation is that we only included the costs from one site, but as all sites had similar characteristics, this limitation is unlikely to be substantial. Thirdly, the study did not differentiate between the costs of counseling and testing those who had a positive HIV test result and those who had a negative result. In addition, linkage to care was self-reported, as we were not able to check self-reported linkage to care against health facility records. We acknowledge that there may be over-reporting. However, linkage to care from the mobile service was similar to that found in another Cape Town study [34]. Generalizability of the results is also acknowledged as a limitation. The study was conducted within a peri-urban area around Cape Town and caution should be exercised when generalizing to other parts of South Africa or other African countries, even where HIV prevalence and incidence is similar. However, the study does provide a unique example of a costing analysis for two CB-HIV testing modalities in a peri-urban setting and thereby contributes to the general body of literature.

## Conclusion

The response to the South African HIV epidemic is largely funded by the government [6]. Contracting NGOs to deliver quality HIV testing services is one solution to the limited government resources available. This study provides insight into the cost of an NGO-led community-based HTS project. It fills a gap in the literature by utilizing detailed and recent cost data to determine the costs of implementing two community-based NGO-led HIV testing modalities (stand-alone and mobile) and the costs associated with realizing key HIV outputs for these two HIV testing modalities in a peri-urban setting. The findings show that due to the higher numbers of people who accessed mobile services, the mean cost per person counseled and tested for HIV was much lower for the mobile modality than for the stand-alone modality. However, due to the lower HIV yield at the mobile services, the cost per person diagnosed and linked to HIV care was lower at the stand-alone modality. Overall, this study highlights (1) the importance of including all applicable costs (including overheads) to ensure an accurate cost estimate that is representative of the full service implementation cost, (2) the direct link between test uptake and mean cost per person tested, and (3) the need for effective linkage to care strategies to increase linkage and thereby reduce the mean cost per person linked to HIV care.

## Additional files

Additional file 1: Categories of core and support personnel included in the study. (PDF 16 kb)

Additional file 2: Examples of costs included in each program component per cost categories per CB-HTS modality. (PDF 40 kb)

## Abbreviations

ART: Antiretroviral therapy
CB-HTS: Community-based HIV testing services
DTTC: The Desmond Tutu TB centre at Stellenbosch University
ELISA: Enzyme Linked Immunosorbent Assay
HIV: Human immunodeficiency virus
HTS: HIV testing services
NGO: non-governmental organization
NHLS: National Health Laboratory Service
UNAIDS: Joint United Nations Programme on HIV/AIDS

## Footnotes

1 The bi-annual audit evaluated client folders randomly sampled from the stand-alone centre and the mobile modality. The audit included a review of client folders to check that all the major aspects of the HIV testing service had been completed and recorded correctly. This included checking; that the client records contained all the necessary demographic and health data, if there was a record of a risk reduction discussion, if family planning needs were assessed and an action noted around the needs, if TB screening was done, if consent for an HIV test was taken, if condoms were offered, if there is a record of referral to an HIV service, evidence that the client had accessed public health services for HIV care and treatment etc. Any gaps in service provision were identified and training with the relevant personnel was implemented to close these gaps, to ultimately ensure that a standardized service was provided across NGOs.
