# Supplementary material for: Cost analysis of two community-based HIV testing service modalities led by a Non-Governmental Organization in Cape Town, South Africa
Source: BMC Health Serv Res. 2017 Dec 2;17:801. doi: 10.1186/s12913-017-2760-8 (PMC5712171; doi:10.1186/s12913-017-2760-8)
Supplement: Supplementary file 1 — Categories of core and support personnel included in the study. (PDF 16 kb) [file 12913_2017_2760_MOESM1_ESM.pdf]

Additional Information – file 1

Categories of core and support personnel included in the study

Categories of core personnel directly involved with service provision in the CB-HTS project.

Personnel category	Total employed for CB-HTS project	Total employed at study site (n=6)	Employed by	Effort
NGO coordinator	5	1	NGO	50%
HIV counsellors	15	3	NGO	100%
Professional nurse	5	1	SU (seconded to NGO)	100%
Enrolled nurse	5	1	SU (seconded to NGO)	100%
Data Clerks	2	N/A	SU	100%

Categories of personnel involved in supporting the CB-HCT project (not involved in direct service provision)

Personnel category	Total personnel providing support (n=10)	Employed by	Effort
Management	3	SU	4%, 50% and 80%
Administration	1	SU	100%
Human resources	2	SU	Both 10%
Data	2	SU	25% and 20%
Driver	1	SU	100%
NGO administrator	5	NGO	20%
