# Supplementary material for: Cost analysis of two community-based HIV testing service modalities led by a Non-Governmental Organization in Cape Town, South Africa
Source: BMC Health Serv Res. 2017 Dec 2;17:801. doi: 10.1186/s12913-017-2760-8 (PMC5712171; doi:10.1186/s12913-017-2760-8)
Supplement: Supplementary file 2 — Examples of costs included in each program component per cost categories per CB-HTS modality. (PDF 40 kb) [file 12913_2017_2760_MOESM2_ESM.pdf]

Additional Information – file 2

Examples of costs included in each program component per cost categories per CB-HTS modality

Cost categories	HCT Modalities	CB-HCT project componenets						
		Admin	Capacity Building	Monitoring & Evaluation	Data	Planning	Direct Services	Overheads
(1) Personnel	Stand-alone	Proportion of core personnel costs	Proportion of core personnel costs	Proportion of core personnel costs	Proportion of core personnel costs	Proportion of core personnel costs	Proportion of core personnel costs	Proportion of support personnel costs
	mobile	Proportion of core personnel costs	Proportion of core personnel costs	Proportion of core personnel costs	Proportion of core personnel costs	Proportion of core personnel costs	Proportion of core personnel costs	Proportion of support personnel costs
(2) Capital Items	Stand-alone	Proportion of a laptop, desktops, printers, office furniture	Proportion of a laptop, desktops, printers, office furniture	Proportion of a laptop, desktops, printers, office furniture	Proportion of a datbase server, laptop, desktops, printers	Proportion of a laptop, desktops, printers, office furniture	Point of care CD4 analyzer, glucometer, scale, blood pressure equipment, proportion of office furniture	None
	mobile	Proportion of a laptop, desktops, printers, office furniture	Proportion of a laptop, desktops, printers, office furniture	Proportion of a laptop, desktops, printers, office furniture	Proportion of database server, laptop, desktops, printers	Proportion of a laptop, desktops, printers, office furniture	Mobile van, tents, point of care CD4 analyzer, glucometer, scale, blood pressure equipment, folding tables, plastic chairs	None
(3) Recurring goods/services	Stand-alone	Proportion of stationery and printing	Proportion of stationery and printing	Proportion of stationery and printing	None	Proportion of stationery and printing	Waste disposal, medical supplies, laboratory costs, HIV rapids test kits, condoms, printing	Proportion of rent, utilities, IT support, telephone, cleaning & sanitary services, NGO admin costs, general maintenance, occupational health insurance, vehicle running costs (support personnel)
	mobile	Proportion of stationery and printing	Proportion of stationery and printing	None	None	Proportion of stationery and printing	Waste disposal, medical supplies, laboratory costs, HIV rapids test kits, condoms, printing, travel (towing caravan), caravan servicing/licencing	Proportion of rent, utilities, IT support, telephone, cleaning & sanitary services, NGO admin costs, general maintenance, occupational health insurance, vehicle running costs (support personnel)
